# Wearable multi-sensing double-chain thermoelectric generator

**DOI:** 10.1038/s41378-020-0179-6

**Published:** 2020-09-07

**Authors:** Dan-Liang Wen, Hai-Tao Deng, Xin Liu, Guo-Ke Li, Xin-Ran Zhang, Xiao-Sheng Zhang

**Affiliations:** School of Electronic Science and Engineering, University of Electronic Science and Technology of China, Chengdu, 611731 China

**Keywords:** Electrical and electronic engineering, Nanoscale devices

## Abstract

Wearable electronics play a crucial role in advancing the rapid development of artificial intelligence, and as an attractive future vision, all-in-one wearable microsystems integrating powering, sensing, actuating and other functional components on a single chip have become an appealing tendency. Herein, we propose a wearable thermoelectric generator (ThEG) with a novel double-chain configuration to simultaneously realize sustainable energy harvesting and multi-functional sensing. In contrast to traditional single-chain ThEGs with the sole function of thermal energy harvesting, each individual chain of the developed double-chain thermoelectric generator (DC-ThEG) can be utilized to scavenge heat energy, and moreover, the combination of the two chains can be employed as functional sensing electrodes at the same time. The mature mass-fabrication technology of screen printing was successfully introduced to print n-type and p-type thermoelectric inks atop a polymeric substrate to form thermocouples to construct two independent chains, which makes this DC-ThEG flexible, high-performance and cost-efficient. The emerging material of silk fibroin was employed to cover the gap of the fabricated two chains to serve as a functional layer for sensing the existence of liquid water molecules in the air and the temperature. The powering and sensing functions of the developed DC-ThEG and their interactions were systematically studied via experimental measurements, which proved the DC-ThEG to be a robust multi-functional power source with a 151 mV open-circuit voltage. In addition, it was successfully demonstrated that this DC-ThEG can convert heat energy to achieve a 3.3 V output, matching common power demands of wearable electronics, and harvest biothermal energy to drive commercial electronics (i.e., a calculator). The integration approach of powering and multi-functional sensing based on this new double-chain configuration might open a new chapter in advanced thermoelectric generators, especially in the applications of all-in-one self-powered microsystems.

## Flexible electronics: energy harvesting and sensing

A wearable thermoelectric device enables energy generation and sensing for health monitoring. Flexible electronic devices are promising candidates for personal health monitoring, and the ideal device would combine multiple functions in a single device. Two of the most important of these functions are energy generation and sensing. In this paper a team from University of Electronic Science and Technology of China led by Xiao-Sheng Zhang reports a thermoelectric-based device that combines these functions. Based on screen printing technology, they prepare n-type and p-type inorganic films onto a flexible polymer substrate, with their device being able to generate a voltage of up to 151 mV driven by a thermoelectric effect. The water sensitivity and temperature sensitivity of silk fibroin contained in the device enables moisture and temperature to be sensed.

## Introduction

As an essential component of the Internet of Things (IoT), wearable mobile electronics are widely used for environmental sensing and human health monitoring by detecting environmental variables and human physical conditions, which has attracted widespread attention from researchers and entrepreneurs^1–6^. However, the limited battery life makes these electronics require frequent recharging, which is a key issue for wearable electronic devices. In recent years, several micro energy harvesting techniques have been developed as candidates to power wearable electronic devices to extend the battery life by converting ambient micro energy into electricity: thermoelectric generators (ThEGs) for thermal energy^7–9^, triboelectric generators (TEGs)^10–12^ and piezoelectric generators (PEGs)^13–15^ for mechanical energy, solar cells for solar energy^16–19^, etc.^20–24^. However, the output of TEGs and PEGs is an alternating current (AC), and mechanical movement is a necessary condition for them. Furthermore, solar cells must work in the presence of light. In comparison, the use of ThEGs is considered to be one of the most promising approaches to power wearable electronic devices due to the properties of continuous direct current (DC) output and lack of the above restrictions^25–30^.

The working mechanism of a ThEG is based on the Seebeck effect^31–33^, and an applied temperature difference results in charge carriers in the thermoelectric material (i.e., electrons in n-type thermoelectric material and holes in p-type thermoelectric material) diffusing from the hot side to the cold side, therefore, a current is generated in the circuit^34–36^. In order for the ThEG to be a wearable power source, high mechanical stability, electrical output stability, and flexibility are required, therefore, conventional physical cutting methods for bulk inorganic thermoelectric materials are improper for wearable ThEGs due to the material properties of rigidity and brittleness. Some organic^37^ or organic-based composite thermoelectric materials^38^ have been proposed to make ThEGs capable to possess flexibility. However, the low output performance of organic-based flexible ThEGs resulting from the high contact resistance has limited their application as a wearable power source. In recent years, a low-cost and mass-fabrication industrial technique called screen printing has been developed to print Bi_2_Te_2.7_Se_0.3_- and Sb_2_Te_3_-based thermoelectric inks onto a flexible substrate to construct high-performance flexible ThEGs^39–41^. Nevertheless, most of the reported wearable ThEGs only serve as power sources for a sensor network and are not sensors themselves. Therefore, for ThEGs to make a real impact in wearable electronics, further improvements in the integration of ThEGs and sensors are needed.

In this work, to overcome the above issues, we propose a wearable double-chain thermoelectric generator (DC-ThEG) that simultaneously realizes the capabilities of thermal energy harvesting and capacitance-based sensing. The low-cost and mass-fabrication industrial technique of screen printing was successfully introduced to print Bi_2_Te_2.7_Se_0.3_ (n-type)- and Sb_2_Te_3_ (p-type)-based thermoelectric inks atop a flexible substrate of polyimide (PI) film to form 10 pairs of thermocouples. By pre-patterning a 150-mesh screen, the 10 pairs of thermocouples were made into two individual thermoelectric leg chains, whose gap was covered with silk fibroin to realize sensing characteristics. An open-circuit voltage of ~151 mV and remarkable mechanical stability and electrical output stability were achieved in this work. Additionally, due to the differential absorption behaviors of silk fibroin to water in different state in the air (i.e., gas and liquid states), the proposed DC-ThEG was demonstrated to possess the ability to detect the existence of liquid-state water in the air. It is worth mentioning that gaseous-state water in the air refers to water vapor, while liquid-state water in the air refers to tiny droplets, which are suspended in the air due to the balance of gravity and buoyancy. Additionally, due to the linear relationship between the dielectric constant of silk fibroin and temperature, the proposed DC-ThEG has the potential to serve as a temperature sensor. Moreover, in the case of wearing, the characteristics of thermoelectricity generation, detecting the existence of liquid-state water in the air and temperature detection can operate well under each other’s working conditions, which was proven by comprehensive experimental comparisons. In addition, to verify the feasibility of the DC-ThEG as a power source, the DC-ThEG was connected to a series-parallel switching circuit to charge twenty-two 2200 μF capacitors to realize a 3.3 V output, and through this method, the DC-ThEG was successfully demonstrated to power a commercial calculator by converting the surface heat of the human body into electricity.

## Methods

### Preparation of the thermoelectric materials

**Fig. 1:**
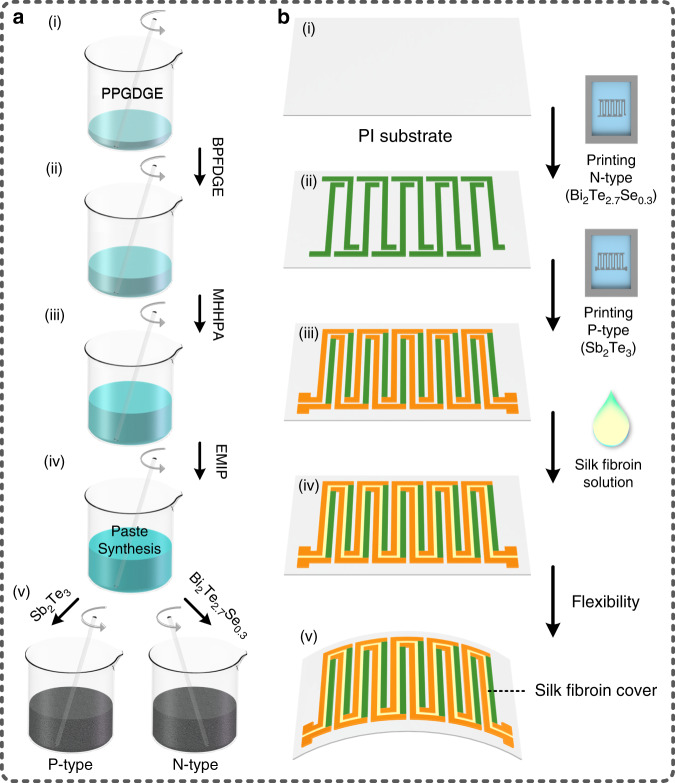
Process flowchart for the proposed wearable multi-functional double-chain thermoelectric generator (DC-ThEG), including the two main steps of the preparation of thermoelectric inks and the printing of the double-chain configuration of the DC-ThEG. **a** Preparation of n-type (Bi_2_Te_2.7_Se_0.3_) and p-type (Sb_2_Te_3_) thermoelectric materials in the form of inks: (i)–(iv) Using the weight ratio of 30.2%:30.2%:38.6%:1%, PPGDGE (polypropylene glycol diglycidyl ether), BPFDGE (bisphenol F diglycidyl ether), MHHPA (methylhexahydrophthalic anhydride, hardener), and EMIP (2-ethyl-4-methyl-1H-imidazole-1-propanenitrile, catalyst) were sequentially mixed to form the paste synthesis. (iv)–(v) Bi_2_Te_2.7_Se_0.3_ powder and Sb_2_Te_3_ powder were evenly mixed with the prepared paste synthesis in weight ratios of 1:5 and 1:6, respectively, to correspondingly form the n-type thermoelectric ink and the p-type thermoelectric ink, respectively. **b** Printing of the proposed DC-ThEG: (i)–(iii) successive printing of the prepared n-type (Bi_2_Te_2.7_Se_0.3_) and p-type (Sb_2_Te_3_) thermoelectric materials on a polyimide (PI) substrate to form complementary double-chain couples by using a screen-printing process, and (iii)-(iv) dispensing of silk fibroin solution atop the gap of the two thermoelectric chains to form a thin silk fibroin layer. It is worth mentioning that there are partially overlapping junctions of the two types of thermoelectric materials instead of traditional metal junctions to ensure lower resistance and simultaneously reduce the structural complexity for better wearable integration

As shown in Fig. 1a, the thermoelectric materials include two types, n-type and p-type, which were prepared by evenly mixing the paste synthesis with Bi_2_Te_2.7_Se_0.3_ powder and Sb_2_Te_3_ powder, respectively. The prepared paste synthesis contained the following three components: 60.4 wt.% liquid epoxy resin, 38.6 wt.% methylhexahydrophthalic anhydride (MHHPA) and 1 wt.% 2-ethyl-4-methyl-1H-imidazole-1-propanenitrile (EMIP). The liquid epoxy resin was prepared by evenly mixing polypropylene glycol diglycidyl ether (PPGDGE) and bisphenol F diglycidyl ether (BPFDGE) in a weight ratio of 1:1, which served as the base solution of the paste synthesis. Then, MHHPA was added to the obtained liquid epoxy, acting as a hardener. Subsequently, EMIP, as a catalyst to decrease the reaction temperature and simultaneously increase the reaction velocity, was added to the mixture solution of liquid epoxy resin and MHHPA to obtain the paste synthesis, as shown in steps (i)–(iv). Finally, Bi_2_Te_2.7_Se_0.3_ powder and Sb_2_Te_3_ powder were added to the obtained paste synthesis in weight ratios of 1:5 and 1:6, respectively, to correspondingly form the n-type Bi_2_Te_2.7_Se_0.3_ ink and p-type Sb_2_Te_3_ ink, as shown in steps (iv)–(v).

### Preparation of silk fibroin solution

The preparation of silk fibroin solution can be summarized into the following steps^42^, as shown Fig. S1 in the Supporting Information file. First, natural *Bombyx mori* cocoons were boiled in 0.02 M sodium carbonate (Na_2_CO_3_) solution for 45 min to remove sericin. Thus, extracted silk fibers were obtained. After being rinsed in deionized (DI) water five times, the silk fibers were dried at 45 °C for 2 h. Subsequently, the dried silk fibers were completely dissolved by immersion in 9.3 M lithium bromide (LiBr) solution at 60 °C for 4 h. To obtain pure silk fibroin solution, the mixed LiBr/silk solution was dialyzed for 48 h using a 3.5 K MWCO dialysis film to remove LiBr ions, and eventually, the silk fibroin solution was further purified through 5 μm microfiltration (Millipore Inc.) three times. It is worth mentioning that an environment below 4 °C was applied during the storage of the obtained silk fibroin solution to decline the gelation effect.

### Fabrication of the proposed DC-ThEG

As shown in Fig. 1b, the fabrication process flow for the proposed DC-ThEG mainly includes four steps. First, a 50 μm-thick polyimide (PI) film was cut into an area of 60 mm × 35 mm, serving as the substrate. Then, the prepared two types of thermoelectric material were successively printed on the PI substrate to form two chain thermocouples by using screen-printing technology, as shown in steps (i)–(ii)–(iii). Specifically, a pre-patterned 150-mesh screen was fixed over the PI substrate, and then, the prepared Bi_2_Te_2.7_Se_0.3_ ink (n-type) was dispensed and evenly scraped by a scraper. Thus, the Bi_2_Te_2.7_Se_0.3_ ink (n-type) was deposited atop the PI substrate through the threads crossing the holes to form n-type thermoelectric legs. To prevent them from being destroyed by the second screen-printing procedure, the printed n-type thermoelectric legs were cured at 90 °C for 30 min. Then, another 150-mesh screen pre-patterned with patterns complementary to those of the previous screen was placed at the same position over the PI substrate as in the last printing procedure, and Sb_2_Te_3_ ink (p-type) was printed atop the PI substrate by the same operations to form p-type thermoelectric legs. Furthermore, a thermal annealing process at 380 °C for 4 h was applied to the two printed thermoelectric materials to recrystallize them, and a thermal annealing process was performed in an oven filled with a N_2_ atmosphere to prevent oxidation of the thermoelectric materials. After annealing, a ThEG with double-chain thermocouples was obtained. Note that the printed thermoelectric legs had partially overlapping areas at the junctions of the two kinds of thermoelectric materials in the long-axis direction, which served to connect the two thermoelectric materials. Compared with the traditional method using copper taps to connect two different thermoelectric legs, partial overlapping printing can achieve a more effective connection between the n-type thermoelectric legs and p-type thermoelectric legs; thus, a lower total resistance of the device was obtained. Additionally, the partially overlapping structures enable lower device complexity for better wearable integration. Finally, the functional sensing material of silk fibroin solution was dropped onto the gap between the two chains of thermoelectric legs and dried at 45 °C for 30 min to form a silk fibroin film to construct the proposed DC-ThEG, as shown in steps (iii)–(iv).

### Tests and measurements

The surface morphologies of the thermoelectric materials were characterized using field emission scanning electron microscopy (SEM, JSM-7600F, JEOL Ltd.). A heating plate was used to supply heat to test the thermoelectric characteristics of the proposed DC-ThEG. The temperatures of the cold side and hot side were measured using a thermometer (GM1312, Benetech Inc.). The thermoelectric output voltages and resistances of the device were measured by using a desktop digital multimeter (DM3068, RIGOL Technology Co., Ltd.). To obtain a stable temperature difference (*ΔT*) for the measurement of fabricated ThEGs, a natural cooling process was designed by setting an initial temperature difference of 60 °C and then turning off the hot plate. Since this natural cooling process is very slow and requires over one hour to cool down to room temperature, at each desired point, *ΔT* remains stable for a specific period long enough for the electrical measurement. When *ΔT* decreased to the desired values, the corresponding output voltages and resistances were recorded, and the above process was repeated three times to calculate the average values to reduce the error. The charging process of capacitors was characterized by a combination of a digital oscilloscope (DS2302A, RIGOL Technology Co., Ltd.) and an electrometer (Keithley 6514, Tektronix Co., Ltd.). To test the characteristic of detecting the existence of liquid-state water in the air, a corresponding test system was established. Specifically, two humidity controllers based on different working principles were employed to supply gaseous- and liquid-state water molecules to simulate air environments with gas-state and liquid-state water, respectively. An LCR meter (E4890A, KEYSIGHT Technology Co., Ltd.) was applied to measure the real-time capacitance change of the DC-ThEG.

## Results and discussion

### Thermal energy harvesting

#### Performance measurement

**Fig. 2:**
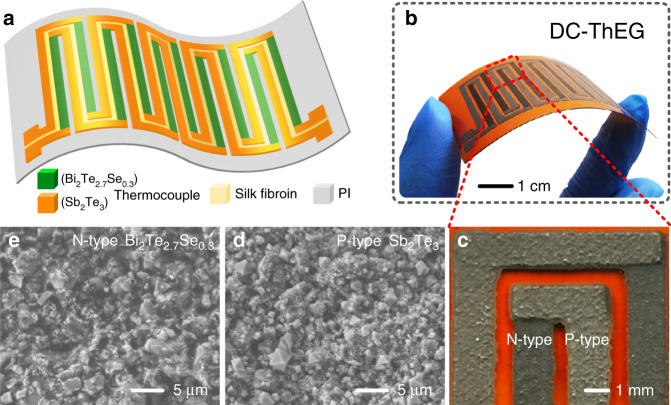
The fabricated double-chain thermoelectric generators (DC-ThEGs) showed remarkable flexibility and uniformity according to the observational characterization based on photographic, optical microscope and scanning electron microscopy (SEM) images. **a** Schematic diagram of the DC-ThEG, which consists of a flexible PI substrate, two thermocouple chains made of Bi_2_Te_2.7_Se_0.3_ (n-type) and Sb_2_Te_3_ (p-type), and a functional chain-gap-covering membrane made of silk fibroin. **b** Photograph of a bent DC-ThEG with a size of 60 mm × 35 mm × 0.2 mm, exhibiting good flexibility. Each individual thermoelectric chain possesses 5 pairs of p-type/n-type thermocouples. **c** High-magnification image showing the junctions of printed p-type/n-type thermocouples. **d**, **e** SEM images of printed n-type and p-type thermoelectric materials. Compared with traditional single-chain thermoelectric generators (ThEGs), this novel double-chain configuration endows ThEGs with the unique feature of simultaneously harvesting thermal energy and realizing multiple sensing functions by utilizing these two chains as electrodes with the help of the functional material covering the gap between them

As shown in Fig. 2a, the proposed DC-ThEG was composed of three main components. First, a PI film was prepared as the substrate, which allows the device to possess good flexibility. Second, double-chain thermocouples made of Bi_2_Te_2.7_Se_0.3_ (n-type) and Sb_2_Te_3_ (p-type) were printed atop the PI film by using a screen-printing process, which serve as thermoelectric legs for thermoelectricity generation and electrodes for multi-functional sensing. Finally, a silk fibroin layer was employed to cover the gap between the two separate thermoelectric leg chains, working as a functional material to detect the existence of liquid-state water in the air and the temperature. Compared with single-chain thermoelectric legs, the structure of double-chain thermoelectric legs allows more functional sensing based on the capacitive effect to be realized (i.e., detecting the existence of liquid-state water in the air and the temperature) while ensuring the power density for thermoelectric generation due to the unchanged number of thermoelectric legs. Figure 2b shows a photograph of a fabricated DC-ThEG with dimensions of 60 mm × 35 mm × 0.2 mm in a bending state, and the demonstrated flexibility reveals the feasibility of the DC-ThEG being a wearable device. The partially enlarged image of the device shown in Fig. 2c shows that the thermocouples were well printed atop the PI substrate. In addition, in this work, the gap between any two adjacent thermoelectric legs, the width of all thermoelectric legs and the length of all thermoelectric legs of the fabricated device were 0.8 mm, 1.5 mm, and 22 mm, respectively. The resistance of two thermoelectric leg chains in series (10 pairs of thermocouples for the entire device) was 1.75 kΩ. The surface morphologies of the two thermoelectric materials of the fabricated device were investigated by using scanning electron microscopy (SEM), as shown in Fig. 2d, e. The compactness and good distribution of the surfaces reveal that the thermoelectric legs were well screen printed atop the PI substrate. In addition, the corresponding average thicknesses of the screen-printed n-type (Bi_2_Te_2.7_Se_0.3_) and p-type (Sb_2_Te_3_) thermoelectric legs were approximately 105 μm and 83 μm, respectively, as shown in Fig. S2 in the Supporting Information file.

**Fig. 3:**
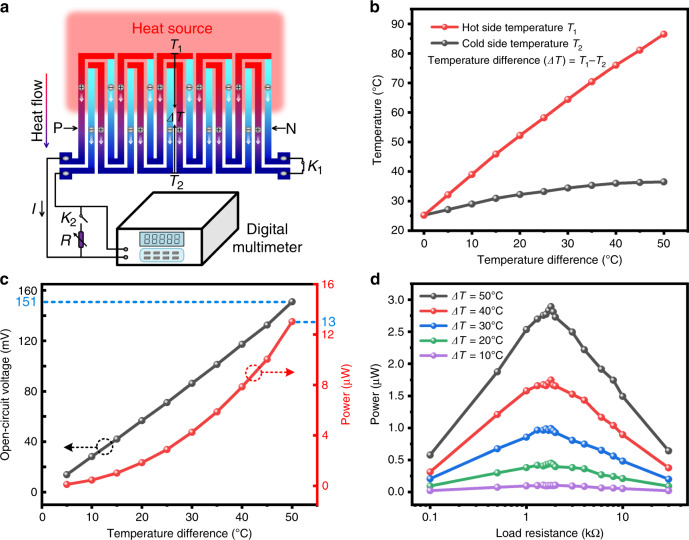
Working mechanism of thermoelectric generation, measurement platform, and thermoelectric output characteristics of the fabricated DC-ThEG. **a** Based on the Seebeck effect, when a heat source is applied to one side of the fabricated DC-ThEG, charge carriers (i.e., electrons in n-type thermoelectric material, Bi_2_Te_2.7_Se_0.3_, and holes in p-type thermoelectric material, Sb_2_Te_3_) diffuse from the hot side to the cold side, resulting in a current, which can be measured by a digital multimeter connected to the two thermoelectric leg chains. Two switches *K*_1_ and *K*_2_ were used to alternate the working states of the device and testing states of the measurement platform. **b** Practical temperature changes of the hot side (*T*_*1*_, red) and cold side (*T*_*2*_, black) of the DC-ThEG versus the increase in the temperature difference (*ΔT*). **c** The open-circuit output voltage (black) and power (red) of the developed DC-ThEG increase as *ΔT* increases (open-circuit state: *K*_*1*_-ON, *K*_*2*_-OFF). **d** Output power to various external load resistors at different *ΔT* (load-connected state: *K*_*1*_-ON, *K*_*2*_-ON). The maximum value of ~2.9 μW was achieved when *ΔT* and the load resistance were 50 °C and 1.8 kΩ, respectively

To verify the feasibility of using the proposed DC-ThEG as a wearable power source for thermal energy harvesting, the comprehensive output performance of the fabricated device was systematically investigated by a series of tests and measurements. Figure 3a illustrates the schematic diagram of the working mechanism based on the Seebeck effect of the proposed DC-ThEG for thermal energy harvesting. When a *ΔT* is applied to the device, charge carriers (i.e., electrons in the n-type thermoelectric material of Bi_2_Te_2.7_Se_0.3_ and holes in the p-type thermoelectric material of Sb_2_Te_3_) diffuse from the hot side to the cold side, resulting in a potential being generated in the circuit, which can be measured by a digital multimeter. Moreover, a switch *K*_*1*_ was used to alternate the working states of the device (i.e., thermal energy harvesting and multi-functional sensing), and a switch *K*_*2*_ was used to alternate the testing states of the measurement platform (i.e., open-circuit state and load-connected state).

The temperature difference is an essential parameter to evaluate the performance of ThEGs, which is defined as the temperature at the hot side minus that at the cold side simultaneously measured by a thermometer. Figure 3b shows the practical temperature rising trends of hot side temperature *T*_*1*_ and cold side temperature *T*_*2*_ when a 90 °C heat source was applied to one side of the device. The hot side temperature *T*_*1*_ rose sharply from 25.2 °C (room temperature) to 86.5 °C, while the cold side temperature *T*_*2*_ rose slightly from 25.2 °C to 36.5 °C. As shown in Fig. 3c, when switch *K*_*1*_ was turned on and switch *K*_*2*_ was turned off, the open-circuit output voltage of the developed DC-ThEG was obtained, which increased linearly with increasing temperature difference *ΔT* applied to the device (i.e., the black curve). A high open-circuit output voltage of ~151 mV and an output power of 13 μW (i.e., the red curve) were achieved when the *ΔT* of the device was 50 °C. Figure 3d shows the output performance of the proposed DC-ThEG for external loads with various resistances from 0.1 kΩ to 30 kΩ at different temperature differences when switches *K*_*1*_ and *K*_*2*_ were turned on. We can observe the following two aspects: First, when the value of the load resistor was constant, the output power to the corresponding load resistor increased with increasing temperature difference of the device. Second, when the temperature difference of the device was constant, the output power to the resistors first showed a rising trend as the resistance of the external load resistors increased from 0.1 kΩ to 1.8 kΩ and then showed a declining trend as the resistance of the external load resistors increased from 1.8 kΩ to 30 kΩ. In other words, when the fabricated device worked as a thermal energy harvester, its matched load was ~1.8 kΩ. In this work, the maximum output power of the fabricated DC-ThEG to external resistors reached ~2.9 μW when the *ΔT* of the device was 50 °C and the load resistor had a matched resistance of 1.8 kΩ. In addition, taking the total area of the thermoelectric legs and junctions into account (i.e., 8.43 cm^2^), the corresponding maximum load output power density was 3.44 μW/m^2^. It is worth mentioning that due to the continuous direct current output property of ThEGs, the fabricated device has the potential to quickly charge capacitors to power wearable electronics, which is demonstrated in the section “The charging property of the DC-ThEG”.

#### Reliability and repeatability measurement

**Fig. 4:**
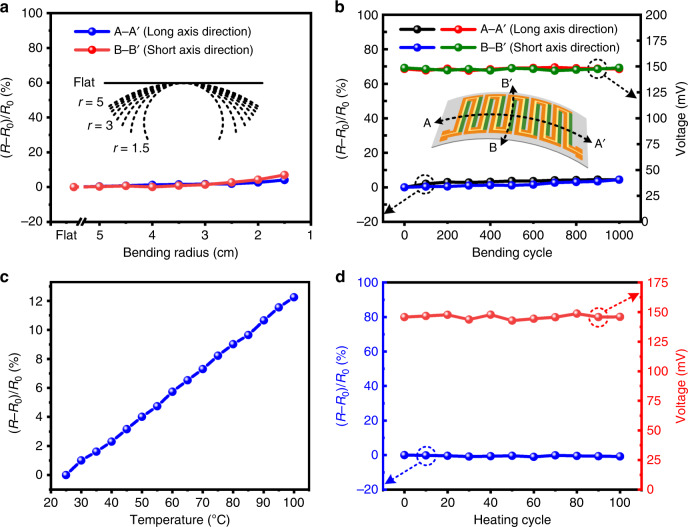
Comprehensive reliability tests of the developed DC-ThEG. **a** Resistance change ratios of the fabricated DC-ThEG in the bending state for various radii from 5cm to 1.5cm, shown in the inset in (**a**), along the long-axis direction (A–A′) and the short-axis direction (B–B′), shown in the inset in (**b**). The fabricated device can be bent to a radius of 3 cm in both directions while the resistance remains almost constant. **b** Resistance change ratios and output voltages (*ΔT* = 50 °C) of the developed DC-ThEG at different bending cycles in both the A–A′ and B–B′ directions, which were remarkably stable even after 1000 bending cycles. **c** Similar to other traditional ThEGs, the resistance of this DC-ThEG increases as the temperature increases, but it is worth mentioning that the increase is linear and stays at a relatively low level with a less than 1% increase per 6 °C. **d** The repeatability of the developed DC-ThEG was tested by 100-cycle heating, which revealed that its resistance and output performance remained steady. In brief, as one of most important factors of sustainable power sources for wearable electronics applications, the reliability and repeatability of the proposed DC-ThEG were proven

For wearable energy harvesting devices, remarkable mechanical reliability, and output repeatability are required. Therefore, a series of reliability and repeatability tests were carried out to systematically evaluate the wearability of the proposed DC-ThEG, as shown in Fig. 4. Figure 4a exhibits the resistance change ratios of a fabricated DC-ThEG in the bending state for various radii from 5 cm to 1.5 cm in both the long-axis (A–A′) and short axis (B–B′) directions. The change ratio of the resistance is defined as *(R−R*_*0*_*)/R*_*0*_, where *R*_*0*_ refers to the initial total resistance of the 10 pairs of thermocouples and *R* refers to the real-time resistance tested under special operation. The inset of Fig. 4a shows an illustration of the various bending radii. When the fabricated DC-ThEG was bent along the long-axis (A–A′) and short axis (B–B′) to a radius of 3 cm, the corresponding resistance change ratios of the 10 pairs of thermocouples were 1.69% and 1.38%, respectively. In other words, the fabricated device can be bent to a radius of 3 cm in both directions while the total resistance remains almost constant. Figure 4(b) shows the resistance change ratios and output performance of the fabricated DC-ThEG after enduring different bending cycles to a radius of 3 cm. After 1000 bending cycles to a radius of 3 cm in the long-axis direction (A–A′) and the short axis direction, the corresponding change ratios of the resistance of the device were 4.41% and 4.46%, respectively, while the output voltages of the device at *ΔT* = 50 °C remained almost consistent with the original value. The inset of Fig. 4b exhibits a bending illustration of the device marked with the bending directions. As shown in Fig. 4c, the resistance of the two chains slightly increased with increasing temperature, resulting from the enhancement of the phonon scattering of charge carriers reducing the mobility of charge carriers^43,44^. The resistance change was approximately linear, with a low rate of a less than 1% increase per 6 °C. In addition, the repeatability of the proposed DC-ThEG was studied by a 100-cycle heating experiment, as shown in Fig. 4d. After 100-cycle heating, both the total resistance of the 10 pairs of thermocouples (returned to room temperature) and the output voltage of the device (*ΔT* = 50 °C) remained highly consistent with the corresponding original values. In summary, the above analysis reveals the remarkable mechanical reliability and output repeatability of the developed DC-ThEG, which make it meet the requirements for being a reliable power source of wearable electronic devices.

### Multi-functional sensing

To improve the integration and functionality of wearable ThEGs, we developed a novel structure of double-chain thermoelectric legs, as shown in Fig. 2a, which makes the ThEG feasible as a capacitance-based sensor while ensuring the generated electricity density. In this work, we used silk fibroin to cover the gap between the two thermoelectric leg chains to serve as the functional component for detecting the existence of liquid-state water in the air and the temperature.

In fact, two-state water (i.e., gas and liquid states) can coexist in the air. The gaseous-state water in the air is water vapor, while the liquid-state water in the air refers to suspended tiny droplets that have a balance between gravity and buoyancy, i.e., fog. Conventional humidity sensors can react to both liquid- and gaseous-state water in the air; therefore, it is very difficult for conventional humidity sensors to judge whether liquid-state water molecules exist in the air. Due to the differential absorption behaviors of silk fibroin for different-state water in the air (i.e., gas and liquid states), the proposed DC-ThEG was demonstrated to be a sensor for detecting the existence of liquid-state water in the air.

**Fig. 5:**
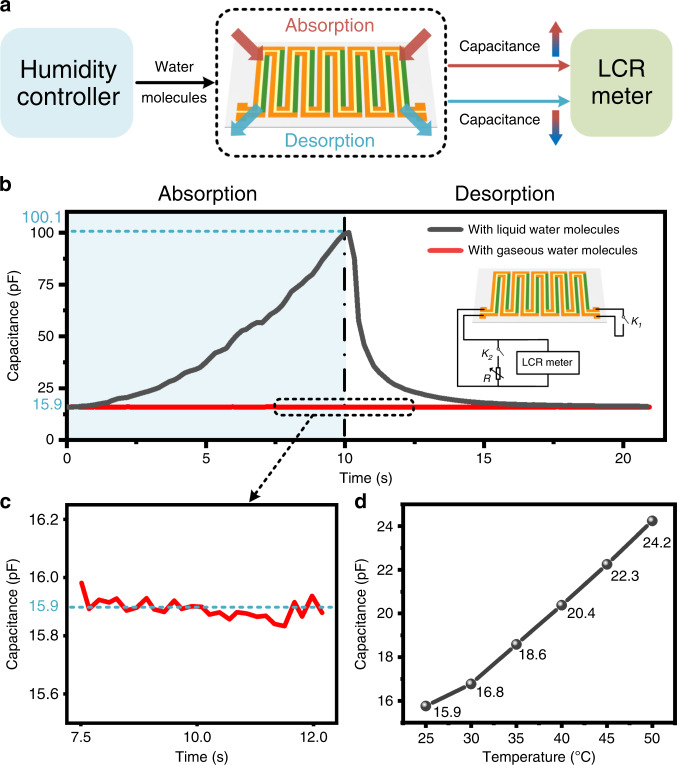
Functional sensing mechanism and measurement of the developed DC-ThEG. **a** When the silk fibroin between the two thermoelectric leg chains absorbs water molecules, its dielectric constant increases, resulting in an increase in the capacitance of the DC-ThEG. In contrast, the desorption of water molecules from the silk fibroin leads to a decrease in the capacitance of the device. A test setup was built to analyse the absorption behaviors of the fabricated device for water molecules in two different states; this setup consists of two humidity controllers to supply liquid- and gaseous-state water, respectively, and an LCR meter to trace the capacitance change in real time. **b** Absorption behaviors of silk fibroin for different-state water in the air (i.e., gas and liquid states). In this function, both switches *K*_*1*_ and *K*_*2*_ were turned off, as shown in the circuit diagram in the inset. The fabricated device exhibited a dramatic capacitance increase from 15.9 pF to 100.1 pF when liquid water molecules were applied for 10 s (black). After the removal of liquid water molecules at 10 s, the capacitance sharply declined. In contrast, the capacitance of the fabricated device exhibited no change (red) after gaseous water molecules were applied for 10 s, except for only normal fluctuations, as shown in (**c**). **d** A good linear relationship between the capacitance and temperature was observed, which provides reasonable evidence verifying the feasibility of using the developed DC-ThEG for temperature sensing

The working mechanism and measurement of the proposed DC-ThEG for the detection of liquid-state water in the air are exhibited in Fig. 5a–c. When the silk fibroin between the two thermoelectric leg chains absorbs water molecules, its dielectric constant increases, resulting in an increase in the capacitance of the DC-ThEG. In contrast, the dielectric constant of the silk fibroin declines when the silk fibroin desorbs water molecules, leading to a decrease in the capacitance of the DC-ThEG. The above working mechanism combined with the differential absorption behaviors of silk fibroin for different-state water in the air (i.e., gas and liquid states) allows the developed DC-ThEG to detect the existence of liquid-state water in the air. In the experiment, a test setup was built to verify this characteristic of the fabricated device, as shown in Fig. 5a. Two humidity controllers based on different working principles were separately used to supply gaseous- and liquid-state water molecules to simulate the corresponding air conditions, and an LCR meter was applied to trace the real-time capacitance change of the proposed DC-ThEG.

When switches *K*_*1*_ and *K*_*2*_ were turned off, the DC-ThEG worked in a capacitance-based sensing mode, as shown in the circuit diagram in the inset of Fig. 5b. The experimental results shown in Fig. 5b exactly validate the differential absorption behaviors of silk fibroin for the two-state water in the air. In the experiment, from 0 s to 10 s, two-state water were separately applied to the device to observe the capacitance changes in the corresponding absorption processes. At 10 s, the supply of water applied was stopped, and the device was removed to normal air conditions to investigate the desorption processes. When the two-state water were separately applied to the DC-ThEG, apparently different capacitance response behaviors of the fabricated device were observed. The capacitance response of the device to gaseous-state water in the air, shown as the red curve in Fig. 5b, remained almost constant in both the absorption and desorption processes. From the enlarged capacitance response waveform shown in Fig. 5c, only ~0.2 pF normal fluctuations were observed before and after the tenth second, revealing no reaction of the fabricated device to gaseous-state water in the air. In contrast, the fabricated DC-ThEG exhibited an intense capacitance increase in response to liquid-state water, as shown by the black curve in Fig. 5b. The capacitance of the DC-ThEG rapidly increased from the initial value of ~15.9 pF to ~100.1 pF within 10 s after the liquid-state water supplied by the humidity controller was applied to it. When the supply of liquid-state water was stopped and the device was removed to normal air conditions, the capacitance of the DC-ThEG rapidly dropped to 25 pF within 2 s due to the large water molecule concentration difference between the device and air environment and then recovered to the initial value ~8 s later. Therefore, the developed DC-ThEG was proven to possess the ability to detect the existence of liquid-state water in the air based on a combination of Fig. 5a–c and the above analysis. This feature also has good repeatability, which can be proven by combining Figs. S3, S4, and the corresponding analysis in the Supporting Information file.

In addition, a change in temperature usually causes a change in the dielectric constant of a dielectric material, i.e., an increase or a decrease, which may be linear or nonlinear. An increase in temperature will intensify the molecular motion of silk fibroin, leading to an increase in the dielectric constant of silk fibroin^45^. In this work, we also studied the impact of temperature on the dielectric constant of the silk fibroin we prepared by observing the change in the capacitance of the device with temperature. As a result, a linear relationship between the capacitance of the DC-ThEG and temperature was observed, as shown in Fig. 5d, which provides powerful evidence demonstrating the feasibility of the developed DC-ThEG functioning as a temperature sensor.

### Interaction between thermal energy harvesting and functional sensing

**Fig. 6:**
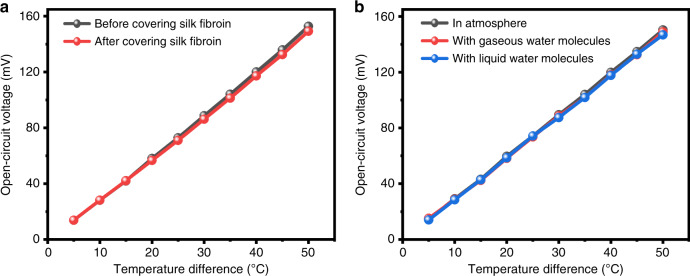
Investigation of the effect of the integration of functional sensing on the thermoelectric output performance of the proposed DC-ThEG. **a** Comparison of the output performances of the device before and after covering the gap between the two thermoelectric leg chains with silk fibroin. A slight decrease in the open-circuit voltage of the device occurred after covering the gap with silk fibroin only in cases of large temperature differences (i.e., *ΔT* > 25 °C). **b** Comparison of the output performances of the device in an atmospheric environment, a gaseous-state water molecule-filled environment, and a liquid-state water molecule-filled environment. In the three environments, the open-circuit voltages of the device had slight differences only in cases of large temperature differences (i.e., *ΔT* > 30 °C). Therefore, in the case of wearing, the proposed DC-ThEG possesses good stability to different-state water molecules

**Fig. 7:**
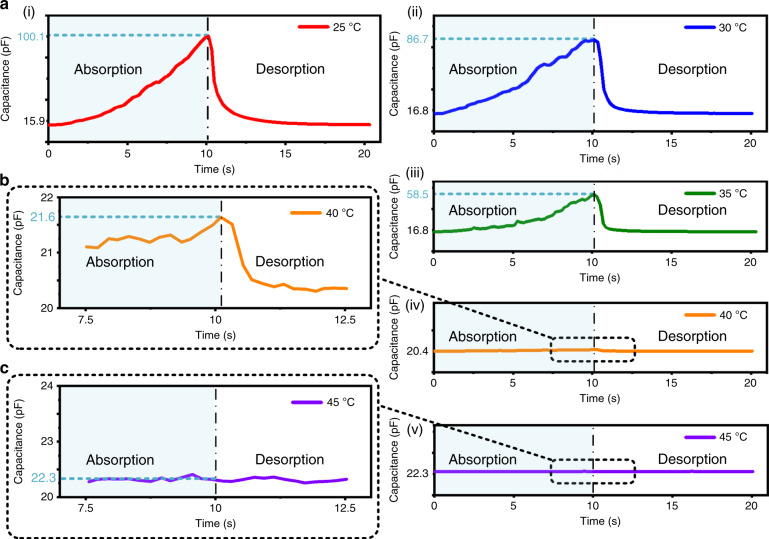
Investigation of the effect of the temperature applied for thermoelectric generation on the functional sensing of detecting the existence of liquid-state water molecules in the air. **a** Response behaviors of the developed DC-ThEG to supplied liquid-state water molecules at different temperatures. From (i) to (v), the DC-ThEG exhibited an apparently weakened capacitance response as the temperature increased. At 40 °C, only a slight capacitance change was observed in **b** the enlarged image of the capacitive response behavior, and at 45 °C, the developed DC-ThEG exhibited no obvious capacitance change except for normal fluctuations in response to liquid-state water molecules, which can be viewed in **c** the enlarged view of the capacitance response waveform

In this work, thermal energy harvesting based on the thermoelectric effect (i.e., the Seebeck effect) and multi-functional sensing based on the capacitive effect were integrated in a single device; however, these characteristics might interact due to the differences between their working conditions, i.e., a temperature difference for power generation and moist air for functional sensing. Therefore, to systematically investigate the interaction between these characteristics, a series of experimental comparisons were carried out, as shown in Figs. 6 and 7. The effect of the silk fibroin cover on the thermoelectric output performance of the proposed DC-ThEG was studied by testing the same device before and after covering the gap between the two thermoelectric leg chains with silk fibroin, as shown in Fig. 6a. The open-circuit voltage of the device after covering the gap with silk fibroin film showed a slight decay only in cases of large temperature differences, such as *ΔT* > 25 °C. In other words, in the case of wearing, the silk fibroin cover had almost no effect on the thermoelectric performance of the device. Moreover, we placed the device in an atmospheric environment, a gaseous-state water molecule-filled environment and a liquid-state water molecule-filled environment and tested the corresponding output voltages to investigate the effect of the two-state water molecules on the output performance of the device. As shown in Fig. 6b, regardless of whether the fabricated DC-ThEG worked in the gaseous-state water molecule-filled environment or the liquid-state water molecule-filled environment, a negligible decay in the open-circuit voltage of the device was observed only in cases of large temperature differences, such as *ΔT* > 30 °C, indicating the stability of the developed DC-ThEG to moisture in thermal power generation, especially in the case of wearing.

In addition, the impact of temperature on functional sensing was studied by observing the capacitance response behaviors of the DC-ThEG for supplied liquid-state water molecules at different device temperatures, as shown in Fig. 7. From the capacitance response waveforms shown in Fig. 7a, the response of the proposed DC-ThEG to supplied liquid-state water molecules gradually weakened as the device temperature increased. When the temperature of the device reached 40 °C, the corresponding capacitance response only showed a slight increase from 20.4 pF to 21.6 pF within 10 s after the liquid-state water supplied by the humidity controller was applied to the device, and the capacitance of the device remained almost consistent when the device temperature reached 45 °C, which can be observed in the enlarged capacitance response waveforms shown in Fig. 7b, c, respectively. Therefore, the characteristic of the fabricated DC-ThEG of detecting the existence of liquid-state water in the air was susceptible to temperature. Fortunately, the surface temperature of human skin and ambient temperature are usually not higher than 40 °C; therefore, in wearing conditions, the developed DC-ThEG possesses a sensitive capacitive response to liquid-state water in the air, enabling it to serve as a detector of liquid-state water in the air.

It is worth mentioning that both thermal energy harvesting and temperature sensing are based on changes in temperature; therefore, there is no mutual influence between them. In other words, the thermal power generation and sensing functions of liquid-state water detection and temperature detection can coexist under each other’s working conditions in the case of wearing.

### The charging property of the DC-ThEG

**Fig. 8:**
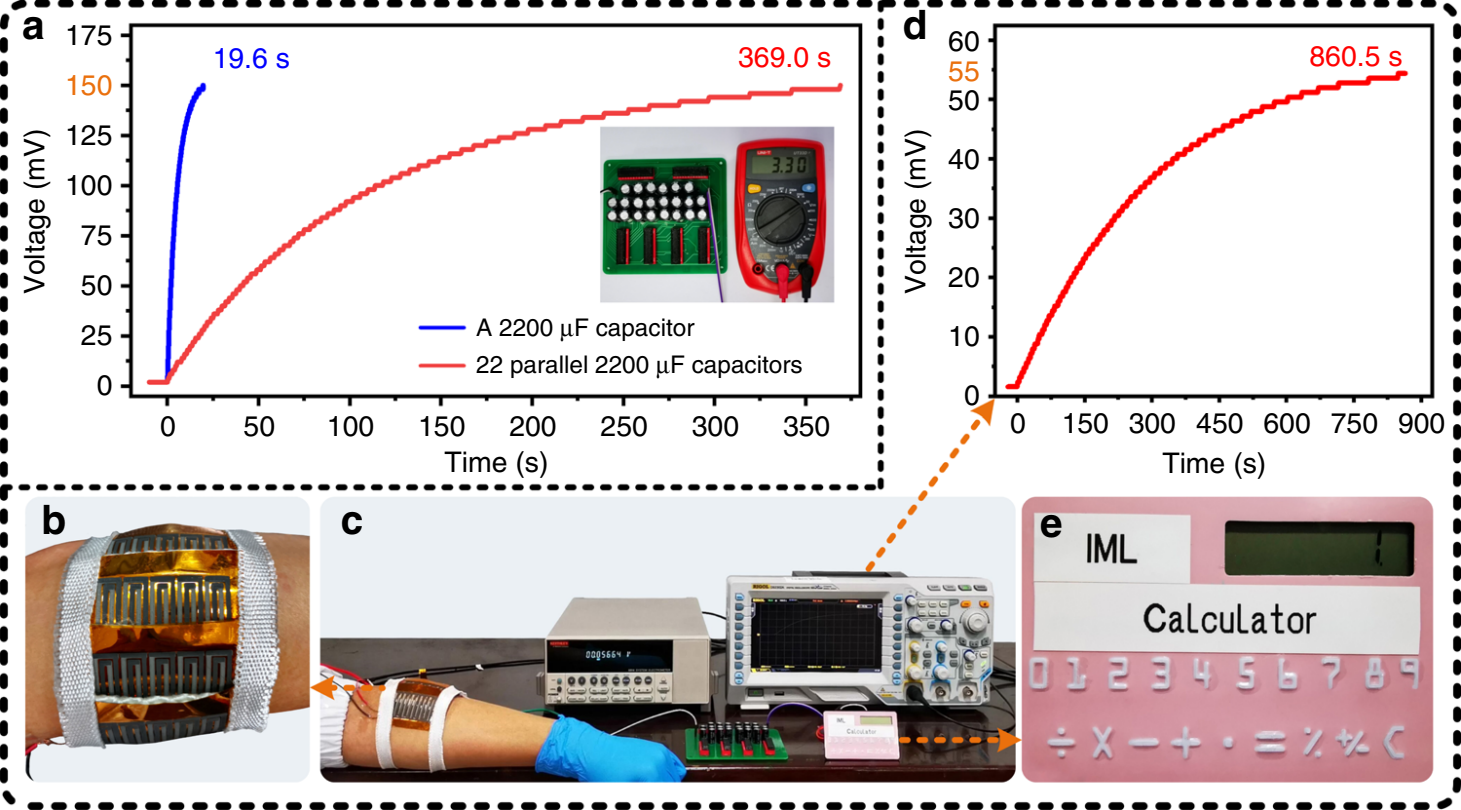
The proposed DC-ThEG was demonstrated to possess an excellent charging capability and to successfully power portable electronics. **a** The times for charging a 2200 μF capacitor and twenty-two parallel 2200 μF capacitors from 0 to 150 mV were 19.6 s and 369.0 s, respectively. (inset) Through a series-parallel switching circuit, a 3.3 V output was obtained, which is commonly used to drive low-power-consumption electronic devices, such as wearable electronics. **b**–**e** Fabricated DC-ThEGs were worn on an arm to harvest human body heat to charge twenty-two parallel 1000 μF capacitors to 55 mV in 860.5 s, and subsequently, a commercial calculator was successfully driven

As a green energy technology, ThEGs are expected to be applied to convert industrial waste heat and human body heat into electrical energy; thus, they are considered a solution for the energy crisis and energy pollution. One of the main parameters for evaluating the performance of a ThEG is the charging capability. In this work, for two application scenarios of high-temperature environments and wearing conditions, we tested the charging property of the proposed DC-ThEG, as shown in Fig. 8. For high-temperature environments, we took *ΔT* = 50 °C as an example and tested the charging capability of the proposed DC-ThEG by charging a 2200 μF capacitor and twenty-two parallel 2200 μF capacitors. As shown in Fig. 8a, the charging times for the capacitor and twenty-two parallel capacitors to be charged from 0 to ~150 mV were 19.6 s and 369.0 s, respectively. It is worth mentioning that the twenty-two 2200 μF capacitors were connected to a series-parallel switching circuit and then to the DC-ThEG. The circuit diagram of the series-parallel switching circuit is shown in Fig. S5 in the Supporting Information file. After the twenty-two parallel capacitors were charged to 150 mV, we turned off the parallel switches and turned on the series switches; thus, a 3.3 V output was achieved, as shown in the inset of Fig. 8a. This 3.3 V output can power most commercial electronic devices, revealing the potential practicability of the proposed DC-ThEG. In addition, to further evaluate the charging capability of the DC-ThEG, several other capacitors with different capacitance values were selected to be charged with the DC-ThEG, i.e., 100 μF, 220 μF, 470 μF, and 1000 μF, as shown in Fig. S6 in the Supporting Information file. It took only 11.9 s for twenty-two parallel 100 μF capacitors to be charged from 0 to 150 mV, verifying the excellent charging capability of the proposed DC-ThEG. Furthermore, to indicate the real impact on the sustainable wearable energy supply, 4 DC-ThEGs were connected in series and worn on a human arm to convert human body heat into electricity to charge twenty-two parallel 1000 μF capacitors, as shown in Fig. 8b, c. It took 860.5 s to charge the twenty-two parallel 1000 μF capacitors to 55 mV, as shown in Fig. 8d, and a more than 1.2 V output was obtained by the series-parallel switching circuit, which can power some low-power-consumption electronic devices, such as the commercial calculator shown in Fig. 8e. The corresponding processes of charging the capacitors and powering the calculator are exhibited in Supplementary Video S1. It is worth mentioning that high silica cloth with a thickness of 1 mm was attached to one side of the devices, serving as a heat insulation layer to ensure a temperature difference. In summary, the above experimental results combined with the results shown in Fig. 5 indicate that the proposed DC-ThEG will have a wide range of applications in the foreseeable future.

## Conclusions

In this work, a wearable double-chain thermoelectric generator (DC-ThEG) was developed to achieve the integration of thermal energy harvesting and capacitance-based multi-functional sensing. The proposed DC-ThEG was fabricated by using a screen-printing process to print Bi_2_Te_2.7_Se_0.3_ (n-type)- and Sb_2_Te_3_ (p-type)-based thermoelectric inks atop a PI substrate to form double-chain thermocouples and by filling the gap between the two chains with silk fibroin to form a functional sensing layer. As a result, the DC-ThEG demonstrated an output capability of 151 mV (*ΔT* = 50 °C), and remarkable electrical output stability and mechanical stability were experimentally confirmed at the same time. Furthermore, the capabilities to detect the existence of liquid-state water in the air and the temperature were successfully verified. The above functions were experimentally proven to coexist in the case of wearing. As an attractive application example, a 3.3 V output was achieved by harvesting thermal energy, which meets the energy supply requirements of most wearable electronics. Moreover, by harvesting human body heat with DC-ThEGs and storing the converted electricity in capacitors, a commercial calculator was successfully driven. In summary, the integration of energy harvesting and multi-functional sensing achieved in this work reveals wide application prospects for the proposed DC-ThEG.

## Supplementary information

Supplementary Video S1

Supporting Information File
