# Supplementary material for: Wearable multi-sensing double-chain thermoelectric generator
Source: Microsyst Nanoeng. 2020 Sep 7;6:68. doi: 10.1038/s41378-020-0179-6 (PMC8433441; doi:10.1038/s41378-020-0179-6)
Supplement: Supplementary file 2 — Supporting Information File [file 41378_2020_179_MOESM2_ESM.docx]

**Supporting Information File**

Wearable Multi-Sensing Double-Chain Thermoelectric Generator

Dan-Liang Wen^1, §^, Hai-Tao Deng^1, §^, Xin Liu^1^, Guo-Ke Li^1^, Xin-Ran Zhang^1^, Xiao-Sheng Zhang^1,*^

^1^School of Electronic Science and Engineering, University of Electronic Science and Technology of China, Chengdu 611731, China

*Corresponding Author: [zhangxs@uestc.edu.cn](mailto:zhangxs@uestc.edu.cn) (XS Zhang)

§These authors contributed equally to this work


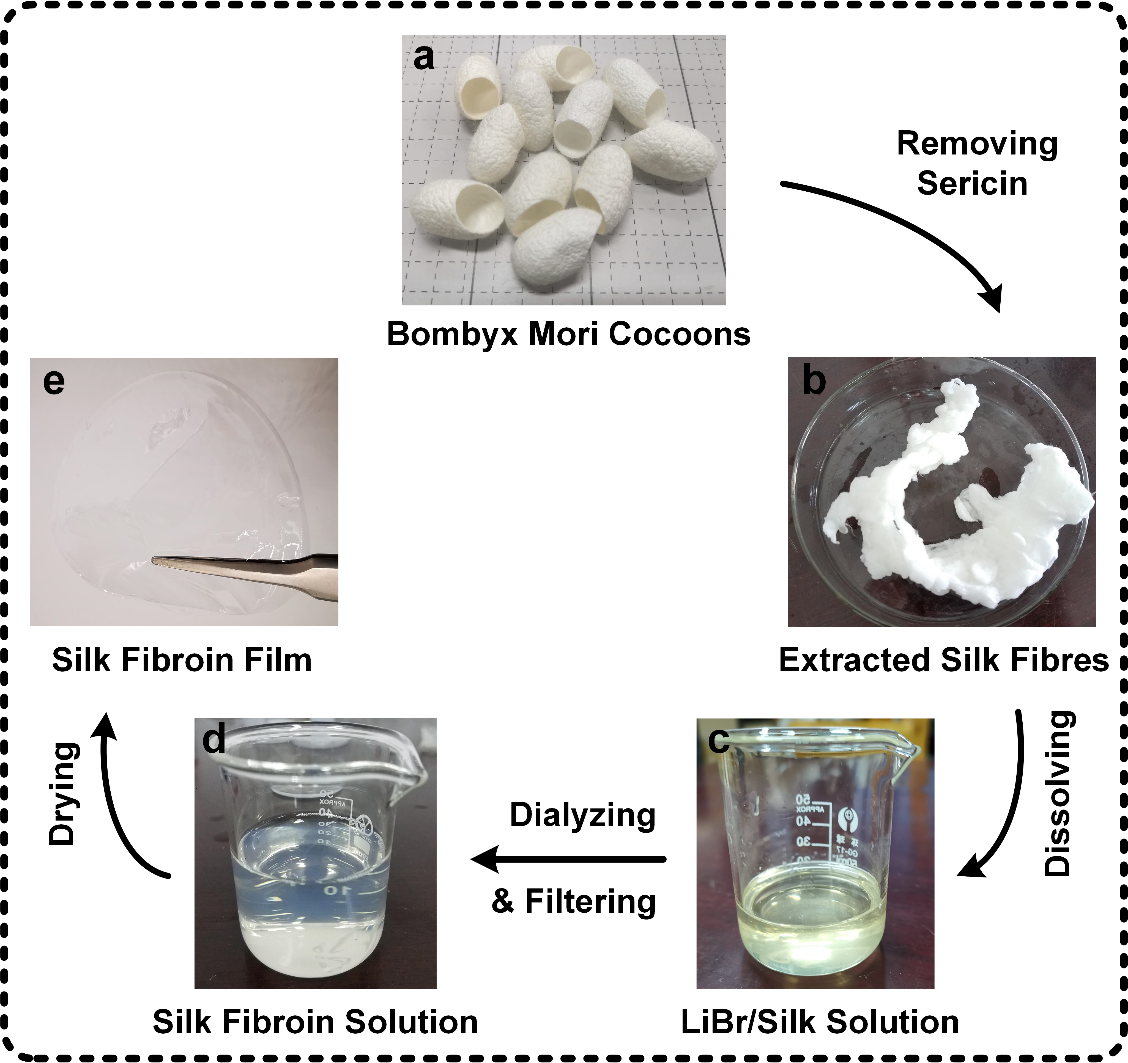


**Figure S1**. Preparation of silk fibroin solution/film. (a) Natural *Bombyx mori* cocoons. (b) Extracted silk fibres prepared by boiling the natural *Bombyx mori* cocoons in the 0.02 M Na_2_CO_3_ solution for 45 minutes to remove sericin. (c) LiBr/silk mixed solution obtained by immersing the extracted silk fibres in 9.3 M lithium bromide (LiBr) solution and baking at 60 ^o^C for 4 hours. (d) Poured silk fibroin solution. The LiBr/silk mixed solution was purified by two steps, dialyzing by a 3.5 K MWCO dialysis film to remove LiBr irons, and then filtering three times through 5 μm microfiltration. (e) A silk fibroin film prepared by drying the silk fibroin in a petri dish.


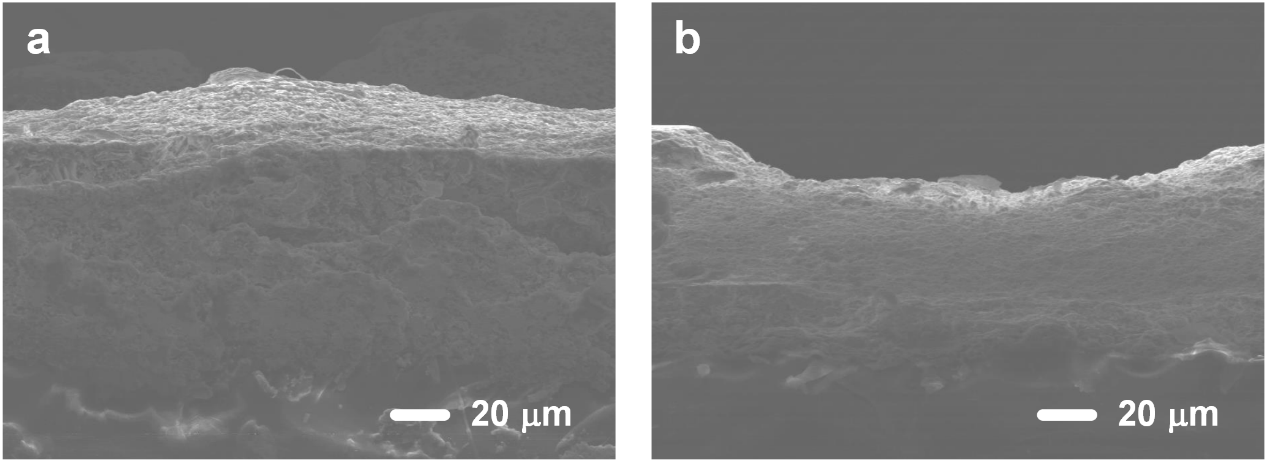


**Figure S2.** The corresponding average thickness of printed (a) n-type thermoelectric material (Bi_2_Te_2.7_Se_0.3_) and (b) p-type thermoelectric material (Sb_2_Te_3_) were approximately 105 μm and 83 μm, respectively. The thickness of thermoelectric materials is one of the key parameters that determine the internal resistance of fabricated device. For thermoelectric generators (ThEGs), devices with smaller internal resistance possess stronger output performance. Therefore, we need to ensure that the thermoelectric materials are thick enough when we print.


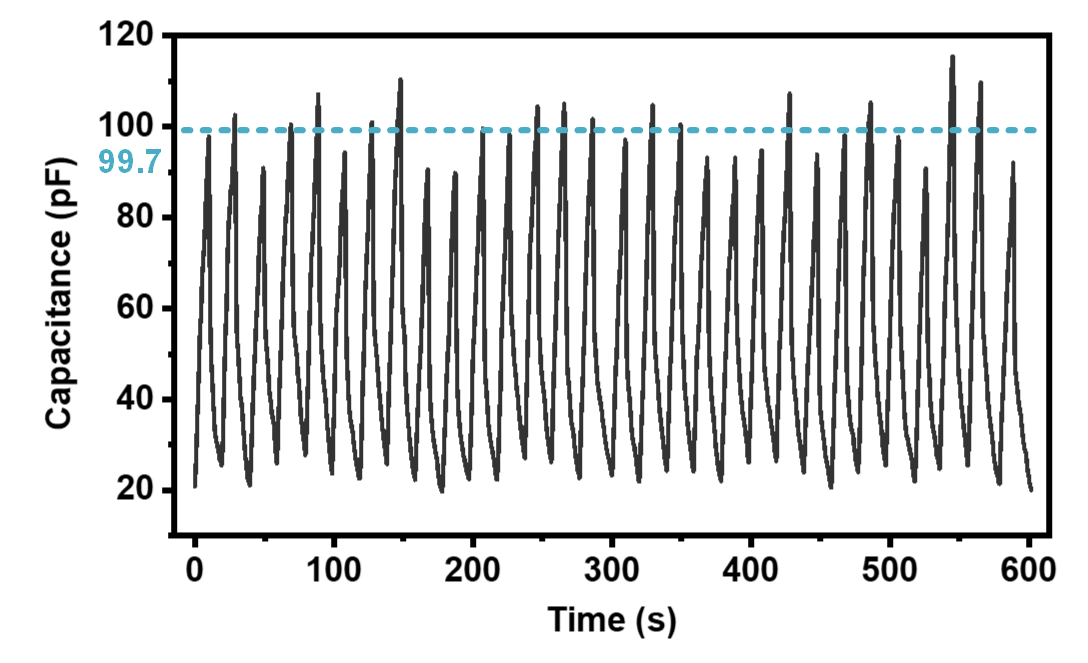


**Figure S3.** Continuous repeatability test of a fabricated double-chain thermoelectric generator (DC-ThEG) to detect the existence of liquid-state water in the air. After 30 repetitive operations of applying liquid-state water molecules to DC-ThEG and stopping supplying after 10 s, the peak values of capacitance response fluctuated within the range of 89.6 pF to 112.7 pF, and the average peak value was 99.7 pF. Considering the aim to detect the existence of liquid-state water in the air, the above response result demonstrated a good repeatability of fabricated device.


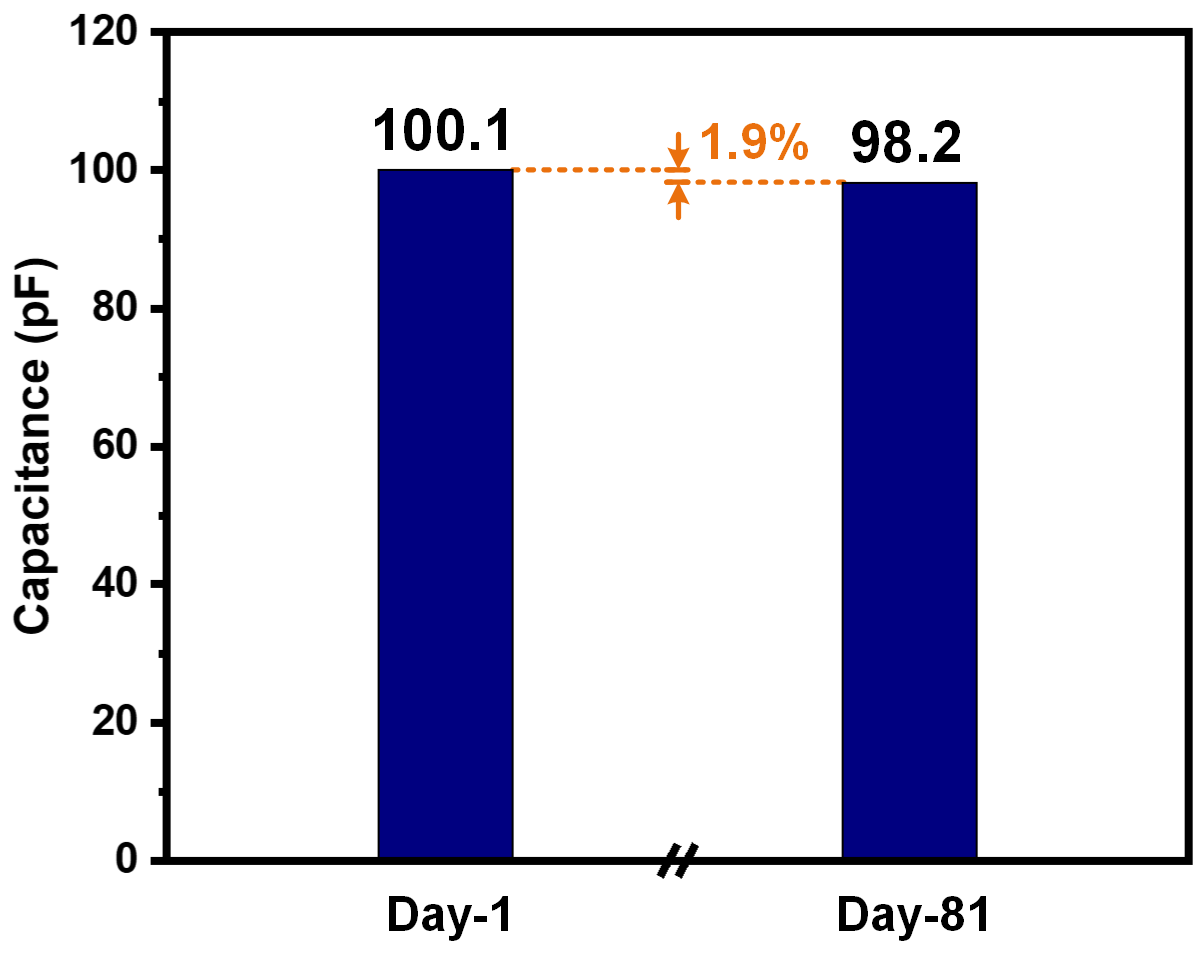


**Figure S4.** Comparison of 80-day sensing behavior and features of the fabricated DC-ThEG. The measured capacitance response of a DC-ThEG after 80 days was 98.2 pF, which only declined by less than 1.9 % compared with the initial value of 100.1 pF. In other word, as for 80-day long-term test, the performance of the DC-ThEG for sensing the existing of liquid-state water in the air kept highly consistent.

**
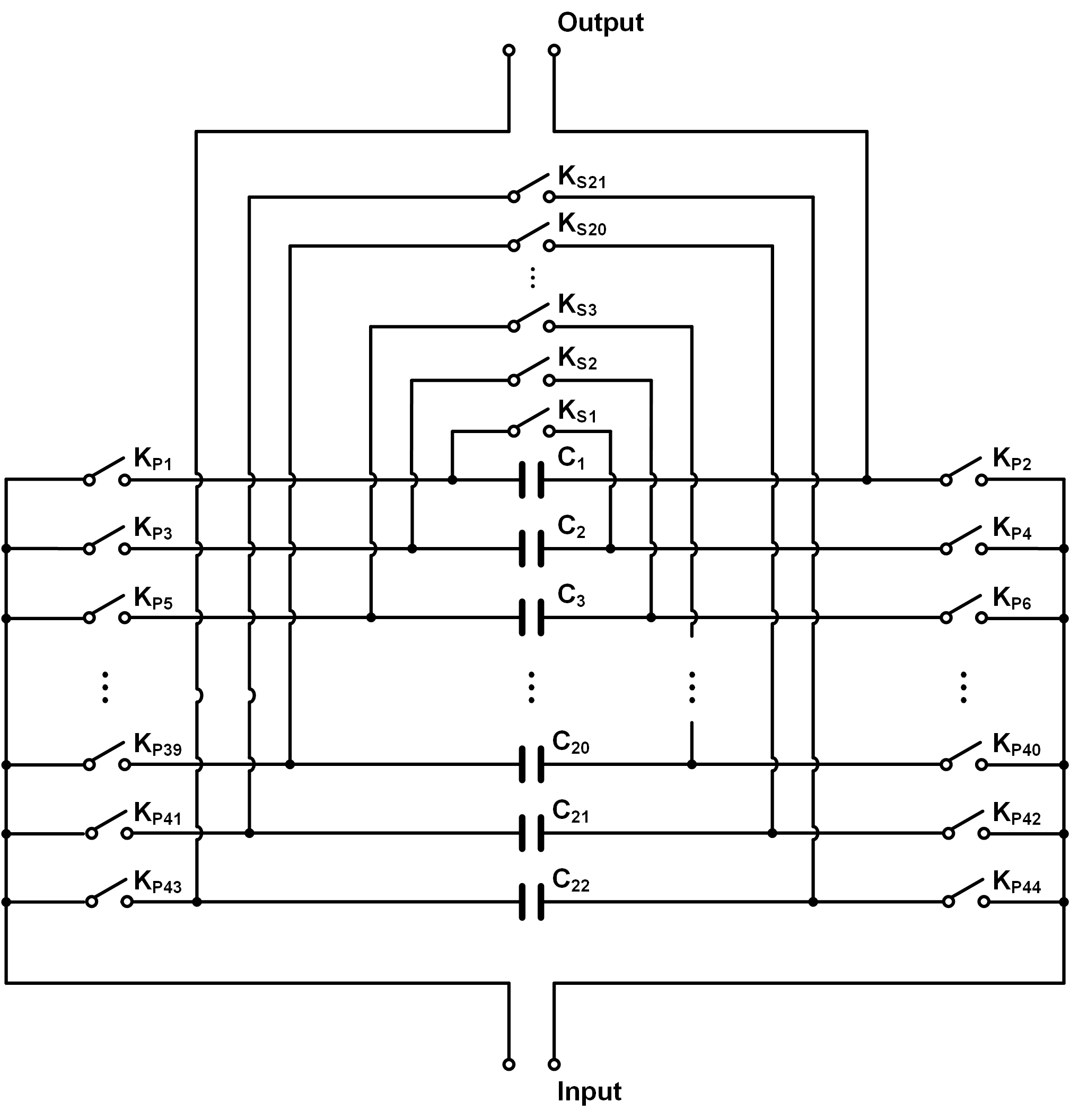
**

**Figure S5.** The circuit diagram of series-parallel switching circuit. When the series switches *K_S_* are turned off and the parallel switches *K_P_* are turned on, the twenty-two capacitors are charged. When the capacitors are charged to the expected voltage *V_E_*, we turn off the parallel switches *K_P_* and turn on the series switches *K_S_*, and a 22 × *V_E_* output is obtained.

**
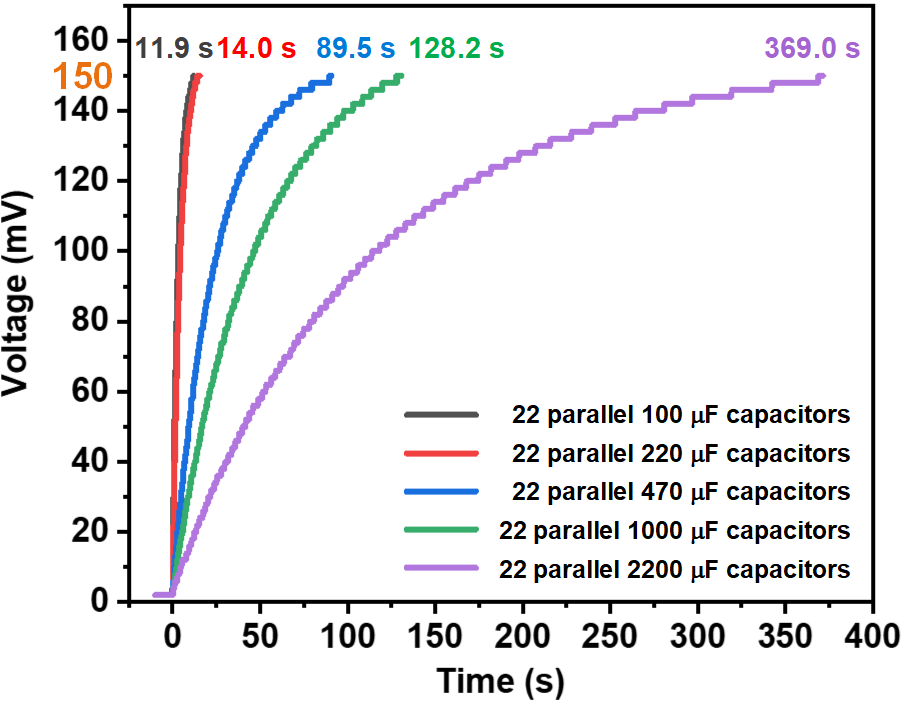
**

**Figure S6.** Comparison of charging rates of different capacitors by using the fabricated DC-ThEG (*ΔT* = 50 °C), i.e. 100 μF, 220 μF, 470 μF, 1000 μF and 2200 μF. Corresponding to twenty-two parallel 100 μF, 220 μF, 470 μF, 1000 μF, and 2200 μF capacitors, the charging times of their voltage increasing from 0 to 150 mV are 11.9 s, 14 s, 89.5 s, 128.2 s, and 369.0 s, respectively. This proved the excellent charging capability of the proposed DC-ThEG.

Supplementary Video:

**Video S1:** 4 DC-ThEGs were connected in series and worn on a human arm to convert human body heat into electricity to charge twenty-two parallel 1000 μF capacitors, and a low-power-consumption calculator was driven by the capacitors.
